# Study on syndrome rules of stagnated heat in liver and stomach of non-erosive reflux disease based on microecology of tongue coating

**DOI:** 10.1097/MD.0000000000031618

**Published:** 2022-11-04

**Authors:** Xinqi Jiang, Hongjie Cheng, Naiwei Zhang, Shanshan Xu, Libao An, Ling Yan, Fang Niu, Boyi Jia

**Affiliations:** a Beijing University of Chinese Medicine, Beijing, People’s Republic of China; b Fangshan Hospital, Beijing University of Chinese Medicine, Beijing, People’s Republic of China.

**Keywords:** a cross-sectional clinical trial, non-erosive reflux disease, stagnated heat in liver and stomach syndrome, tongue coating microecology

## Abstract

At present, the pathogenesis of non-erosive reflux disease (NERD) is still unclear, and proton pump inhibitors are the main treatment drug. However, the effect is limited. Traditional Chinese medicine treatment of NERD has advantages. Stagnated heat in liver and stomach syndrome is the most important traditional Chinese medicine syndrome type of this disease. Tongue diagnosis is an important basis for the diagnosis of stagnated heat in liver and stomach syndrome. The microecology of tongue coating suggests the occurrence and development of disease. The purpose of this study aims to clarify the regular changes of tongue coating microecology in stagnated heat in liver and stomach syndrome of NERD.

**Methods and analysis:**

This is a cross-sectional clinical trial. This study is divided into NERD stagnated heat in liver and stomach syndrome group, qi stagnation, and phlegm obstruction syndrome control group and normal control group, with 20 cases in each group. Tongue coating samples will be collected from 3 groups, and 16SrRNA gene sequencing technology will be used to detect the genome of tongue coating flora in patients with NERD with stagnated heat in liver and stomach syndrome, control group with qi stagnation and phlegm obstruction syndrome and normal control group. The main outcome measures are the distribution, diversity, and richness of the tongue flora in patients and healthy controls.

**Discussion:**

The results of this study will clarify the internal relationship between NERD stagnated heat in liver and stomach syndrome and the microecological changes in tongue coating.

## 1. Introduction

Gastroesophageal reflux disease (GERD) refers to a type of disease in which gastroduodenal content flows back into the esophagus, causing esophageal symptoms and complications,^[1]^ the incidence of this disease is gradually increasing,^[2]^ which has a great impact on the quality of life of patients. GERD can be divided into reflux esophagitis, non-erosive reflux disease (NERD), and Barrett’s esophagus. A study in the United States showed that the incidence of GERD was about 26%, and about 70% of the patients were NERD.^[3]^ Recent studies show that the incidence of NERD in China also accounts for about 70% of GERD,^[4]^ and its incidence is increasing year by year, NERD has become 1 of the hot spots and difficulties in research of digestive system diseases.^[5]^

NERD refers to reflux disease with typical gastroesophageal reflux symptoms but no visible mucosal damage under endoscopy, also known as endoscopic negative reflux disease.^[6]^ In Roman IV, the newly added “reflux hypersensitivity” also had no mucosal damage, but was distinguished from NERD due to physiological acid reflux sensitivity and normal esophageal acid exposure. Currently, proton pump inhibitors are often used as the preferred drugs for the treatment of NERD, but only about 50% of patients with NERD can be cured.^[7]^ Long-term use of proton pump inhibitor is prone to complications,^[8]^ and the risk of recurrence after withdrawal.^[9]^

A large number of clinical studies have shown that Chinese medicine is flexible in the treatment of NERD based on syndrome differentiation, with few side effects, and has obvious advantages in effectively alleviating NERD-related symptoms, improving anxiety and depression, reducing the recurrence rate, and improving living standards.^[10]^ Treatment based on syndrome differentiation is the key to the traditional Chinese medicine (TCM) treatment of NERD. However, there is no material basis for TCM syndrome formation and identification in clinical practice, and the mechanism of disease occurrence is not yet clear.

At present, a large number of studies on the syndrome type of NERD show that stagnated heat in liver and stomach syndrome is the most important TCM syndrome type of NERD, accounting for 32.2% to 32.6% of all types, accounting for the first.^[11]^ The latest consensus of experts on TCM diagnosis and treatment of GERD (2017) also ranked stagnated heat in liver and stomach syndrome as the first syndrome type.^[12]^ Therefore, a systematic study of stagnated heat in liver and stomach syndrome is an important strategy for the treatment of NERD. Tongue diagnosis is 1 of the unique diagnostic methods in TCM and is an important basis for the diagnosis of NERD of stagnated heat in liver and stomach syndrome. Microecology is an important method of modern scientific research on tongue coating, and also an important extension of tongue diagnosis.^[13]^ At present, the research on the microecology of tongue coating has been gradually carried out, but there is no clinical study on the regularity of the microecology of tongue coating in NERD stagnated heat in liver and stomach syndrome, which deserves further exploration and research in order to clarify the law.^[14]^

Therefore, on the basis of previous studies, we will collect tongue coating specimens from NERD patients with stagnated heat in liver and stomach syndrome, patients with qi stagnation and phlegm obstruction syndrome, and normal control group, using 16SrRNA gene sequencing technology to analyze the diversity and richness of tongue coating flora among the 3 groups, and find the different bacteria genus among the 3 groups, to explore the changes of stagnated heat in liver and stomach syndrome and tongue coating microecology of NERD. The purpose of this study is to further clarify the regular microecological changes of tongue coating in the stagnated heat in liver and stomach syndrome in NERD, to provide a microecological basis for clarifying the essence of stagnated heat in liver and stomach syndrome in NERD, and to provide a new idea and method for interpreting the theory of “tongue diagnosis” by using modern language.

## 2. Methods

### 2.1. Ethics

The trial is designed according to the “Ethical Review of Biomedical Research Involving Human Subjects” issued by Ministry of Health of the People’s Republic of China, CFDA “Code of Quality Management for Drug Clinical Trials (2020),” “Guiding Principles for Ethical Review of Drug Clinical Trials (2010),” and WMA “Declaration of Helsinki.” The implementation of this project has been reviewed and approved by the Ethical Review Committee of Fangshan Hospital of Traditional Chinese Medicine, Beijing University of Chinese Medicine for Boyi Jia (8 Sep 2020, approval number: FZY LK-2020-013); Dr Hongzhang, director of the committee, is the authorized representative under subtitle ethics approval and consent (Ethical Approval Document-Chinese version and Ethical Approval Document-English version). Written informed consent will be obtained from each subject and his/her guardians.

### 2.2. Study design

**Figure 1.**
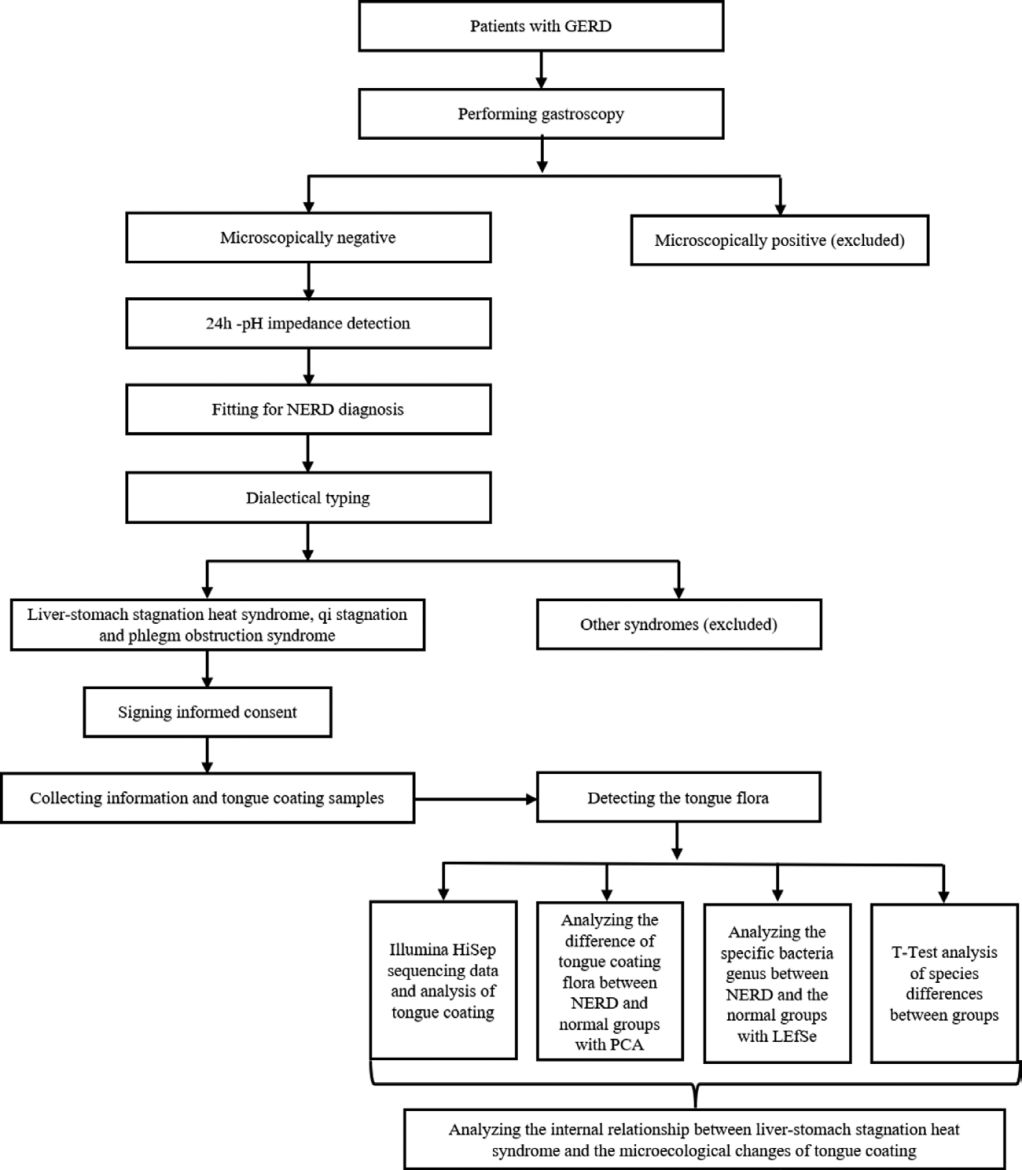
Study design.

This trial will be a cross-sectional clinical trial. The aim of this study is to clarify the regular changes of tongue coating microecology in stagnated heat in liver and stomach syndrome of NERD, providing a microecological basis for the essence of stagnated heat in liver and stomach syndrome of NERD, and provide new ideas and methods for interpreting the theory of “tongue diagnosis” in modern language by studying the difference of tongue coating microecology in the control group and normal control group of NERD with stagnated heat in liver and stomach syndrome and qi stagnation and phlegm obstruction syndrome (Fig. 1). This report will be compiled according to the SPIRIT 2013 Checklist and the STARD statement.

### 2.3. Recruitment

This study is divided into NERD patients with stagnated heat in liver and stomach syndrome, qi stagnation, and phlegm obstruction syndrome control group and normal control group, with 20 cases in each group. The source of the case is outpatient and inpatient patients and healthy volunteers from Department of Spleen and Stomach, Fangshan Hospital of Beijing University of Chinese Medicine from October 1, 2020 to September 31, 2022. Participants are informed of details about the study which are purpose, duration, procedures, and key contacts, as well as risks and potential benefits. Participants may withdraw their consent for any reason without any consequences at any time.

### 2.4. Diagnostic criteria

1. Diagnostic criteria for Western medicine: refer to the Chinese Gastroesophageal Reflux Disease Expert Consensus Opinion (2014) formulated by the Chinese Gastroesophageal Reflux Disease Consensus Opinion Expert Group of the Chinese Gastroenterology Society of the Chinese Medical Association and the *Modern Diagnosis of GERD: the Lyon Consensus* published in 2018.
2. Diagnostic criteria for TCM: refer to the Consensus opinions of TCM Diagnosis and Treatment experts on Gastroesophageal reflux Disease (2017) formulated by the spleen and Stomach Diseases Branch of China Association of TCM, and formulate the scale of stagnated heat in liver and stomach syndrome, qi stagnation and phlegm obstruction syndrome combined with clinical practice (Table 1).

**Table 1:** General information of stagnated heat in liver and stomach syndrome and qi stagnation and phlegm obstruction syndrome.

Syndrome	Stagnated heat in liver and stomach syndrome
Symptoms	Primary symptoms: ① against acid; ② heartburn Secondary symptoms: ① burning pain after sternum; ② epigastric burning pain; ③ full abdominal distension; ④ belching or backfeeding; ⑤ irritability; ⑥ easy hunger
Tongue pulse	Tongue nature red moss yellow or yellow greasy; wiry pulse or thready and slippery pulse
Syndrome	qi stagnation and phlegm obstruction syndrome
Symptoms	Primary symptoms: ① throat discomfort like phlegm stem; ② chest discomfort Secondary symptoms: ① belching or regurgitation; ② dysphagia; ③ hoarseness; ④ cough in the middle of the night
Tongue pulse	White and greasy tongue coating; thready and slippery pulse

### 2.5. Identification of syndrome type

Have 2 primary symptoms and 2 secondary symptoms, refer to tongue pulse.

### 2.6. Eligibility criteria

#### 2.6.1. Inclusion criteria of stagnated heat in liver and stomach syndrome and qi stagnation and phlegm obstruction syndrome.

1. Western medicine diagnosis is consistent with NERD.
2. TCM syndrome differentiation is stagnated heat in liver and stomach syndrome, qi stagnation, and phlegm obstruction syndrome.
3. Those aged between 18 and 65.
4. Voluntary participation and signing of informed consent.

#### 2.6.2. Exclusion criteria of stagnated heat in liver and stomach syndrome and qi stagnation and phlegm obstruction syndrome.

1. Women who are pregnant or trying to become pregnant and women who are breastfeeding.
2. Allergic constitution and allergic to multiple drugs.
3. Patients with serious primary diseases of liver, kidney, hematopoietic system, endocrine system, and mental illness.
4. Serious illness requiring emergency treatment.
5. Participants in other clinical trials within 3 months.

#### 2.6.3. Inclusion criteria for normal control group.

1. Healthy persons (18–65 years old) of TCM dialectically belong to peaceful constitution, and their body mass index is between 18.5 and 23.9. The diagnostic criteria of peaceful constitution refer to the Classification and Determination of TCM Constitution issued in 2009.
2. No infectious diseases or inflammation after medical examination.

#### 2.6.4. Exclusion criteria for normal control group.

1. Those who have hepatitis, syphilis, HIV, schistosomiasis, and other known infectious diseases.
2. Those who have a history of gastroesophageal reflux disease.
3. Those who have acute gastroenteritis within the last 6 months.
4. Those who have taken antibiotics in the past 3 months.
5. Those who have been to an epidemic area in the last 6 months.
6. Those who have a high-risk life history such as yeyou in the past 6 months.
7. Those who have a history of gastrointestinal bleeding in the last 3 months.
8. Those who have had constipation in the last 3 months.
9. Those who have used laxatives and weight-loss drugs in the past 3 months.
10. Those who have taken immunosuppression such as hormones in the last 3 months.
11. Those who have gastrointestinal polyposis, malignant tumor.
12. Those who have chronic diseases such as diabetes, hypertension, and coronary heart disease.
13. Those who have liver cirrhosis and autoimmune diseases.
14. Those who have chronic obstructive pulmonary disease.
15. Those who have severe food allergies.

### 2.7. Implementation plan

High-throughput sequencing 16SrRNA method will be used to detect the tongue coating flora, and the specific steps are as follows:

#### 2.7.1. Image acquisition of tongue coating.

After the patient sits still for 5 minutes, his face will be placed on the ZMT-1A TCM camera. After the placement, his tongue will be extended as far as possible, and the image will be taken and saved for later use.

#### 2.7.2. Tongue coating collection method.

After morning rises, without brushing your teeth and gargling, we will use a sterile swab to roll the brush forward along the root of the tongue to take the coating 10 times, 2 times in total. We will immerse the sterile swab after brushing into a centrifuge tube containing 1 mL phosphate buffer and place it in 4 centrifuges for 5000r for 5 minutes. The centrifuge tube will be removed, the supernatant is discarded, and the precipitation is retained and stored in the −80 refrigerator for later use.

#### 2.7.3. Illumina HiSep sequencing.

Total microbial DNA will be extracted from the sampled tongue coating, and PCR amplification (including denaturation, annealing, and extension) will be performed on the V4 hypervariable region of the 16SrRNA gene of the extracted bacterial DNA, and 2% agarose electrophoresis will be used for detection. Finally, sequencing will be performed using the Illumina HiSep platform.

### 2.8. Data management and statistical analysis

#### 2.8.1. Collecting WeChat registration.

It includes the doctor’s side and the patient side. After the patient side completes the filling and submission, the doctor can refer to it from the doctor’s side, and the statistician will conduct the next statistical analysis based on the data collected by the doctor’s side.

#### 2.8.2. Fill in the CRF form on site.

Screened subjects fill in the clinical information collection form; clinical investigation should be carried out in a quiet and comfortable environment without interference; black pen shall be used to fill in the clinical information collection form; the observation form must be filled in true, accurate and clear, and must not be altered at will. If mistakes need to be corrected, a horizontal line should be drawn in the middle, and the name of the modifier and the modification time should be signed; the CRF table shall be properly kept and placed, and shall not be discarded at will.

#### 2.8.3. Database establishment and data review.

EpiData3.0 will be used to establish the database, and data entry will be carried out independently by 2 people. In case of inconsistency, the original data should be traced and processed by more than 2 experts in the research group. After data entry, a second inspection will be conducted, and 2 other people will modify it until the 2 databases are exactly the same. After data entry is completed, the data will be locked and submitted to the test statistician for statistical analysis.

### 2.9. Statistical analysis

After all data is exported by EpiData3.0, Illumina high-throughput sequencing platform will be used for data analysis and statistics. PCA will be used to analyze the difference in tongue coating flora between NERD patients and the normal group, and the LEfSe method will be used to analyze the specific bacteria genus between the normal group and NERD patients. The measurement data will be described in the form of mean ± standard deviation. A paired sample *t* test will be used for those in line with normal distribution, and a non-parametric test is used for those in line with normal distribution. For counting data, it will be described by the number of cases (percentage), as well as chi-square test, Fisher’s exact probability method, Wilcoxon rank-sum test, etc. All statistical tests adopt 2-sided tests, and *P* < .05 is considered to be statistically significant.

### 2.10. Quality control and quality assurance

#### 2.10.1. Bias analysis.

In the design of this study, possible biases in the study are considered as follows: ① at the beginning of the study, there is selection bias due to the lax inclusion criteria of the tested population; ② in view of possible information bias caused by defects in the methods of measuring exposure and outcome, this study intends to establish relevant SOP and strictly train researchers, and carry out quality control on the inclusion criteria of the tested population, and try to select new cases, use objective data, and pay attention to the way of questionnaire questioning and investigation techniques to avoid selection bias and information bias.

#### 2.10.2. Quality control.

##### 2.10.2.1. Quality control and personnel training.

Invite experts to optimize the scheme, formulate relevant Sops, and train the participants according to the requirements of Sops.

##### 2.10.2.2. Qualifications of researchers.

1. Clinical intervention implementation personnel: those qualified physicians and relevant clinical work experience.
2. Clinical evaluators: those with relevant clinical research experience.
3. Data entry personnel: those with a medical background, familiar with routine computer operators.
4. Quality inspector: those with relevant working experience.

### 2.11. Confidentiality

This trial will fully comply with the relevant provisions of data protection legislation. All appropriate and necessary precautions will be taken to keep medical data and personal information permanently confidential.

## 3. Discussion

The pathogenesis of NERD is still unclear, and its pathogenesis is characterized by multiple factors. Among them, hypersensitivity of esophageal viscera, abnormal gut-brain, decreased pressure of lower esophageal sphincter, decreased ability of esophageal profile clearance, impaired esophageal mucosal barrier, attack of reflux, correlation with Helicobacter pylori, and genetic factors are all current research objects.^[15]^ Although the pathogenesis of NERD is complex, it is generally recognized that the esophageal mucosal barrier is damaged by acid and other gastric reflux substances due to the hypersensitivity of esophageal viscera and the abnormality of gut-brain.^[16,17]^ Currently, the treatment of NERD is still dominated by drugs, while endoscopic treatment and surgical treatment are not widely used and the efficacy is not exact. Symptom relief and improvement of quality of life are still the main treatments direction for NERD.

There is no direct name for NERD in TCM. According to the symptoms, NERD belongs to the categories of “spitting acid” and “swallowing acid.”^[18]^ A large number of clinical studies have shown that TCM is flexible in the treatment of NERD based on syndrome differentiation, with few side effects, and has obvious advantages in effectively alleviating NERD related symptoms, improving anxiety and depression, reducing recurrence rate, and improving living standards.^[19,20]^ According to TCM theory, when the mood is not free, liver and gallbladder lose at catharsis, can cross spleen and stomach, and mood is not free for long.^[21]^ It smolders into fire, liver, and gallbladder fire is inverse, causing stomach gas to go up inverse, the occurrence is this disease. A large number of studies on the syndrome type of NERD have shown that stagnated heat in liver and stomach syndrome is the most important TCM syndrome type of NERD, accounting for 32.2% to 32.6% of all types, accounting for the first place.^[22,23]^ The latest consensus of experts on TCM diagnosis and treatment of GERD (2017) also ranked it as the first syndrome type.^[24]^

Tongue diagnosis can judge the location of disease, the nature of disease, and the ebb and flow of good and evil, and provide a basis for syndrome differentiation. “Clinical Tongue Test Method” says: “According to the tongue, the deficiency and the excess are divided, but the deficiency and the excess are not satisfactory; According to the tongue, yin and yang are divided, and yin and yang are not absurd; According to the tongue, it is divided into 5 internal organs and matched with the main prescription, while the internal organs are not concealed and the main prescription is not mistaken.^[25]^ Therefore, different pathogenic pathogens are reflected in the tongue and their manifestations are also different. In the consensus opinions of TCM diagnosis and treatment experts for GERD (2017) edition,^[24]^ tongue images of different syndrome types are completely different, with obvious differences. For example, stagnated heat in liver and stomach syndrome is red tongue and yellow moss.^[26]^ Tongue coating, as an important part of tongue diagnosis, is often used to distinguish the rise and fall of vital qi, the deficiency, and reality of Zang and Fu, the cold and heat of evil qi, the depth of the disease, to judge the severity of the disease, to infer the prognosis of the disease, and to guide clinical medication, which has important clinical significance.^[27]^

Professor Wei Si, a famous expert in microecology in China, once predicted that “microecology is likely to become a golden key to open the door to the mystery of TCM.^[28]^ Further study on the microorganisms of tongue coating will provide new ideas for further understanding the relationship between disease and the change of tongue coating.^[29]^ Due to the self-cleaning function of the tongue, the tongue coating microecology of normal people plays a normal role, its microecological environment is stable, the total amount of bacteria is small and the species is single, and the epithelial cells of the tongue grow and differentiate normally without obvious inflammation, which constitutes the basis for the formation of the tongue image of thin white coating.^[30]^ Relevant studies have confirmed that changes in tongue flora caused by diseases can also lead to changes in tongue image, that is, different bacterial community structures may respond to different tongue images.^[31]^

In this study, tongue coating specimens from patients with stagnated heat in liver and stomach syndrome, patients with qi stagnation and phlegm obstruction syndrome, and normal control group will be collected. 16SrRNA gene sequencing technology will be used to analyze the diversity and richness of tongue coating flora of the 3 groups, find the different bacteria genera among the 3 groups, explore the changes in stagnated heat in liver and stomach syndrome and tongue coating microecology of NERD, and provide a new idea and method for interpreting the theory of “tongue diagnosis” by using modern language.

However, our study has limitations. The limitation concerns the fact that we will only recruit tongue coating specimens from NERD patients with stagnated heat in liver and stomach syndrome and qi stagnation and phlegm obstruction syndrome. The regularity of microecological changes in tongue coating in other NERD syndromes has not been explored.

Therefore, a clinical trial about the regularity of microecological changes in tongue coating in different NERD syndromes is still needed.

## Author contributions

**Conceptualization:** Boyi Jia.

**Funding acquisition:** Boyi Jia.

**Methodology:** Naiwei Zhang.

**Project administration:** Boyi Jia.

**Resources:** Libao An.

**Supervision:** Hongjie Cheng.

**Validation:** Naiwei Zhang.

**Visualization:** Fang Niu.

**Writing – original draft:** Xinqi Jiang, Shanshan Xu, Libao An, Ling Yan.

**Writing – review & editing:** Boyi Jia.
